# Development of a scoring system with multidimensional markers for fibrosing interstitial lung disease

**DOI:** 10.1038/s41598-022-16382-1

**Published:** 2022-08-20

**Authors:** Shenyun Shi, Lulu Chen, Xiaoqin Liu, Min Yu, Chao Wu, Yonglong Xiao

**Affiliations:** 1 Department of Respiratory and Critical Care Medicine, Nanjing Drum Tower Hospital, Clinical College of Nanjing Medical University, Nanjing, 210008 Jiangsu China; 2 Department of Respiratory and Critical Care Medicine, Nanjing Drum Tower Hospital, the Affiliated Hospital of Nanjing University Medical School, No. 321 Zhongshan Road, Nanjing, 210008 Jiangsu China; 3 Department of Infectious Diseases, Nanjing Drum Tower Hospital, Clinical College of Nanjing Medical University, No. 321 Zhongshan Road, Nanjing, 210008 Jiangsu China

**Keywords:** Biomarkers, Diseases, Rheumatology, Risk factors

## Abstract

Fibrosing interstitial lung disease (ILD) can cause high mortality and sensitive evaluation of fibrosing ILD could be critical. The aim of this study is to develop a scoring system to predict prognosis of fibrosing ILD. 339 patients with fibrosing ILD were enrolled as a derivation cohort. Cox multiple regression analysis indicated that smoking history (HR  =  3.826, *p*  =  0.001), age(HR  =  1.043, *p*  =  0.015), CEA(HR  =  1.059, *p*  =  0.049),CYFRA21-1(HR  =  1.177, *p*  =  0.004) and DLCO% predicted (HR  =  0.979, *p*  =  0.032) were independent prognostic factors for fibrosing ILD. The clinical scoring system for fibrosing ILD was established based on the clinical variables (age [A], CEA and CYFRA21-1 [C], DLCO% predicted [D], and smoking history [S]; ACDS). The area under the receiver operating characteristic curve (AUROC) of the scoring system for predicting prognosis of fibrosing ILD was 0.90 (95%CI: 0.87–0.94, *p* < 0.001). The cutoff value was 2.5 with their corresponding specificity (90.7%) and sensitivity (78.8%). To validate the value of ACDS score levels to predict the survival of patients with fibrosing ILD, 98 additional fibrosing ILD patients were included as a validation cohort. The log-rank test showed a significant difference in survival between the two groups(ACDS score < 2.5 and ACDS score ≥ 2.5) in validation cohort. The independent risk factors for mortality in patients with fibrosing ILD are higher CEA, higher CYFRA21-1, smoking history, lower DLCO%predicted at baseline and older age. ACDS is a simple and feasible clinical model for predicting survival of fibrosing ILD.

## Introduction

Interstitial lung diseases (ILDs) are a group of heterogeneous lung diseases with pulmonary alveolar unit inflammation or interstitial fibrosis that are associated with substantial morbidity and mortality. The causation of ILD includes idiopathic and specific etiology including autoimmune disease, vasculitis, drugs, tumors and occupational or environmental exposure^1,2^. Idiopathic pulmonary fibrosis (IPF) is one of a family of idiopathic interstitial pneumonias characterized by usual interstitial pneumonia (UIP) in high-resolution computed tomography (HRCT) and pathology^3^. IPF has a poor prognosis with median survival from the time of diagnosis approximately 3 years^4^. Fibrosing ILDs other than IPF, such as connective tissue disease (CTD) associated ILD, including ILD associated with rheumatoid arthritis (RA-ILD), systemic sclerosis (SSc-ILD) and polymyositis/dermatomyositis are also known to have progressive disease behaviors similar to IPF^5–7^. Therefore, it is important to recognize the risk factors associated with poor prognosis in patients with fibrosing interstitial lung disease.

Several biomakers has been reported to be as diagnostic and prognostic biomarkers of fibrosing ILD, including Krebs von den lugen-6 (KL-6), Surfactant proteins A and D (SP-A and SP-D), serum interleukin 6 (IL-6) levels and tumor markers^8–10^. However, the relationship between proportion of each serum marker and fibrosing ILD is not clear. Therefore, in this study, we retrospectively studied the clinical characteristics of patients with fibrosing ILD and established a novel model to better guide personalized therapeutic choices in persons.

## Materials and methods

### Study subjects

**Figure 1:**
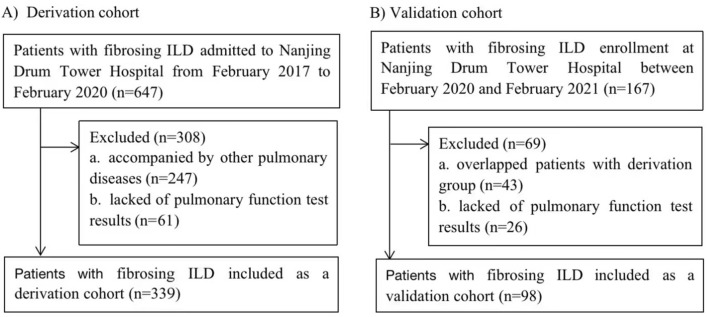
Flow diagram describing the selection of the study population.

We retrospectively reviewed 647 patients who were diagnosed of fibrosing ILD (IPF and CTD-associated UIP) from inpatient of the department of respiration of Nanjing Drum Tower Hospital from February 2017 to February 2020. Overall, 308 patients were excluded based on exclusion criteria. A total of 339 patients were analyzed as a derivation cohort(Fig. 1A). To validate the value of clinical scoring system to predict the survival of patients with fibrosing ILD, a validation cohort was performed which consisted of 98 patients with fibrosing ILD who were admitted to the department of respiration of Nanjing Drum Tower Hospital between February 2020 and February 2021(Fig. 1B). Patients with incomplete data were excluded. Exclusion criteria for all fibrosing ILD subjects were: (1) subjects had combined pneumonia, lung malignancy, or other pulmonary diseases; (2) subjects lacked of pulmonary function test results; (3) subjects of validation cohort overlapped with derivation cohort. We analyzed demographic features, clinical characteristics, lung function parameters and therapy. Survival status was determined by reviewing the medical records or telephone follow-ups until February 2021.

This study was consented by Ethics Committee of Nanjing Drum Tower Hospital. The Ethics Committee waived the need for informed consent as the study was retrospective and the data were analyzed anonymously.

### Methods

The diagnosis for IPF was mainly based on the criteria from An Official ATS/ERS/JRS/ALAT Clinical Practice Guideline^3^. The diagnosis of CTD-ILD referred to the published guideline^11^. Clinical information at admission was collected including demographics, smoking history. Pulmonary function tests including forced vital capacity (FVC), FVC% predicted, diffusion capacity for carbon monoxide (DLCO), and DLCO% predicted were extracted for analysis. All subjects had UIP pattern on chest HRCT as defined by the guidelines from the American thoracic society and the European respiratory society^3,11^.

### Statistical analysis

Data were expressed as mean ± standard deviation (SD). t-Test or the Mann–Whitney U test was used for continuous variables. Categorical variables were compared by Chi-square test. The independent prognostic role of variables were evaluated by Cox proportional hazard analysis. Receiver operator characteristic (ROC) analyses were performed to calculate area under the ROC curve (AUC) of markers for predicting the prognosis of fibrosing ILD. The Kaplan–Meier method was used to assess survival curves with GraphPad Prism version 7 (Graph Pad Software Inc., La Jolla, CA, USA). The log-rank test was used to evaluate the statistical significance of differences between the higher ACDS score and lower ACDS score groups. Statistical analyses were performed using SPSS18.0 statistical software. Statistical significance was considered at the 0.05 levels.

### Ethics approval and consent to participate

The study was conducted according to the Declaration of Helsinki. This study was approved by Ethics Committee of Nanjing Drum Tower Hospital, the Affiliated Hospital of Nanjing University Medical School.The Ethics Committee of Nanjing Drum Tower Hospital, the Affiliated Hospital of Nanjing University Medical School waived the need for informed consent as the study was retrospective and the data were analyzed anonymously.

## Results

### Baseline clinical characteristics of fibrosing ILD patients

The baseline clinical features of subjects with IPF (n  =  132) and CTD-ILD characterized by UIP on HRCT (n  =  207) were summarized in Table 1. Male gender, older age were more common in the IPF group (*p* < 0.001 and *p* < 0.001, respectively). Smoking history was similar. Red blood cell distribution width(RDW) levels, serum total bilirubin (TBIL) and direct bilirubin (DBIL) also differed between the two groups (*p*  =  0.008, *p* < 0.001 and *p*  =  0.001, respectively). Patients with CTD-ILD characterized by UIP on HRCT had a higher DLCO% predicted level compared with the IPF patients.

**Table 1:** Baseline clinical features in the derivation cohort.

Variables	UIP (n = 339)	IPF (n = 132)	CTD-UIP (n = 207)	*p* value
Gender (M/F)	183/156	115/17	68/139	< 0.001
Smoking history (Y/N)	60/279	31/101	29/178	0.026
Age (years old)	62.49 ± 11.24	68.27 ± 8.71	58.80 ± 11.13	< 0.001
WBC count (× 10^9)	7.01 ± 2.07	7.11 ± 1.91	6.94 ± 2.16	0.46
RDW(%)	13.63 ± 1.25	13.42 ± 1.03	13.76 ± 1.35	0.008
PLT(× 10^9)	211.68 ± 71.57	190.62 ± 73.37	225.11 ± 67.21	< 0.001
TBil(umol/l)	8.99 ± 3.28	9.81 ± 3.32	8.48 ± 3.16	< 0.001
DBil(umol/l)	2.68 ± 1.22	2.96 ± 1.23	2.50 ± 1.18	0.001
LDH (U/L)	265.18 ± 77.13	254.52 ± 63.34	271.98 ± 84.20	0.031
B cells (× 10^9)	0.237 ± 0.161	0.244 ± 0.148	0.233 ± 0.169	0.550
NK cells (× 10^9)	0.263 ± 0.197	0.329 ± 0.229	0.222 ± 0.160	< 0.001
CEA (ng/ml)	2.61 ± 2.89	3.20 ± 2.21	2.24 ± 3.17	0.003
CYFRA21-1 (ng/ml)	4.36 ± 2.14	4.58 ± 2.04	4.22 ± 2.19	0.131
NSE (ng/ml)	16.58 ± 5.56	16.69 ± 6.74	16.51 ± 4.68	0.772
PaO2/FiO2 ratio	367.20 ± 75.55	370.36 ± 86.27	364.76 ± 66.32	0.551
FVC% predicted	67.10 ± 17.29	66.72 ± 17.17	67.35 ± 17.41	0.746
FEV1% predicted	73.50 ± 18.44	73.25 ± 17.37	73.65 ± 19.13	0.847
DLCO% predicted	52.69 ± 21.43	49.47 ± 19.02	54.74 ± 22.32	0.027

WBC  =  white blood cell; RDW  =  red blood cell distribution width; PLT  =  platelet; TBil  =  total bilirubin; DBil  =  direct bilirubin; LDH  =  lactate dehydrogenase; NK cells  =  Natural killer cells; CEA  =  carcinoem-bryonic antigen; CYFRA21-1  =  cytokeratin 21–1; NSE  =  neuron specific enolase; PaO2/FiO2  =  oxygenation index; FVC  =  forced vital capacity; FEV1  =  forced expiratory volume; DLCO  =  diffusing capacity for carbon monoxide.

### Constructing a scoring system for predicting prognosis of fibrosing ILD

According to the final follow-up data, 339 fibrosing ILD patients were divided into survivors group (n = 259) and decedents group (n = 80). As was shown in Table 2, there was no difference in the proportion of CTD-UIP and IPF among the survivors group and decedents group (*p* = 0.072). Cox proportional hazards models were used to examine the influence of variables on the prognosis of patients with fibrosing ILD. The multivariate cox regression analysis showed that smoking history (HR = 3.826, *p* = 0.001), age (HR = 1.043, *p* = 0.015), carcinoem-bryonic antigen (CEA) (HR = 1.059, *p* = 0.049), cytokeratin 21–1(CYFRA21-1) (HR = 1.177, *p* = 0.004) and DLCO%predicted (HR = 0.979, *p* = 0.032) were independent prognostic factors for fibrosing ILD (Table 3).

**Table 2:** Comparison between survivors and decedents in fibrosing ILD patients of the derivation cohort.

	Survivors (n = 259)	Decedents (n = 80)	*p* value
fibrosing ILD(CTD-UIP/IPF)	165/94	42/38	0.072
Age (years old)	58.95 ± 10.73	63.58 ± 11.19	0.001
CEA (ng/ml)	2.54 ± 2.17	2.86 ± 4.45	0.537
CYFRA21-1 (ng/ml)	4.34 ± 1.97	4.40 ± 2.62	0.837
DLCO% predicted	58.69 ± 21.17	50.84 ± 21.21	0.004

**Table 3:** Prognostic factors for survival by univariate and multivariate Cox regression models in fibrosing ILD patients of the derivation cohort.

Variables	Univariate Cox model			Multivariate Cox model		
	HR	95.0% CI	*p* value	HR	95.0% CI	*p* value
CTD-UIP(Y)	2.234	1.428–3.496	< 0.001	1.857	0.835–4.134	0.129
Gender	1.376	0.883–2.144	0.159	0.369	0.133–1.024	0.056
Smoking history	5.096	3.279–7.920	< 0.001	3.826	1.686–8.683	0.001
Age (years old)	1.069	1.045–1.093	< 0.001	1.043	1.008–1.079	0.015
WBC count	1.163	1.054–1.282	0.003	1.008	0.843–1.206	0.926
RDW	1.391	1.216–1.591	< 0.001	1.075	0.837–1.381	0.572
PLT	1.000	0.997–1.003	0.765	0.999	0.994–1.003	0.591
TBil	0.964	0.897–1.035	0.310	0.890	0.775–1.023	0.101
DBil	1.015	0.853–1.207	0.871	1.260	0.808–1.966	0.308
LDH	1.003	1.001–1.006	0.002	1.002	0.997–1.006	0.493
B cells	0.710	0.180–2.797	0.624	1.733	0.348–8.634	0.502
NK cells	1.652	0.572–4.772	0.354	2.173	0.417–11.324	0.357
CEA	1.084	1.046–1.123	< 0.001	1.059	1.000–1.122	0.049
CYFRA21-1	1.374	1.290–1.463	< 0.001	1.177	1.053–1.316	0.004
NSE	1.051	1.015–1.087	0.004	1.015	0.970–1.061	0.520
PaO2/FiO2 ratio	0.993	0.990–0.997	< 0.001	0.998	0.993–1.002	0.302
FVC% predicted	0.955	0.940–0.970	< 0.001	1.010	0.956–1.066	0.732
FEV1% predicted	0.970	0.957–0.983	< 0.001	0.965	0.918–1.015	0.171
DLCO% predicted	0.949	0.937–0.960	< 0.001	0.979	0.959–0.998	0.032

CTD-UIP  =  connective tissue disease-usual interstitial pneumonia; WBC  =  white blood cell; RDW  =  red blood cell distribution width; PLT  =  platelet; TBil  =  total bilirubin; DBil  =  direct bilirubin; LDH  =  lactate dehydrogenase; NK cells  =  Natural killer cells; CEA  =  carcinoem-bryonic antigen; CYFRA21-1  =  cytokeratin 21–1; NSE  =  neuron specific enolase; PaO2/FiO2  =  oxygenation index; FVC  =  forced vital capacity; FEV1  =  forced expiratory volume; DLCO  =  diffusing capacity for carbon monoxide.

The accuracy of independent prognostic factors for predicting the survival of fibrosing ILD was then evaluated by Receiver Operating Characteristics (ROC) analysis. The area under the ROC curve for CYFRA21-1 in predicting the survival of fibrosing ILD was 0.85 (95% CI, 0.80–0.90; *p* < 0.001). The prediction ability for smoking history, age, CEA and DLCO%predicted were listed in Table 4. Then, we constructed a simple clinical scoring system for predicting survival of fibrosing ILD with the variables of smoking history, age, CEA, CYFRA21-1 and DLCO%predicted (Table 5).

**Table 4:** Comparisons of ROC curve analysis for predicting the survival of fibrosing ILD patients.

	AUC(95%CI)	*p* value	Cut-off value	Sensitivity	Specificity
Smoking history	0.69(0.61, 0.76)	< 0.001	–	–	–
Age (years old)	0.69(0.63, 0.76)	< 0.001	66.5 years old	64.6%	66.3%
CEA	0.62(0.55, 0.70)	< 0.001	2.3 ng/ml	60.8%	61.6%
CYFRA21-1	0.85 (0.80, 0.90)	< 0.001	4.3 ng/ml	88.6%	74.4%
DLCO% predicted	0.84(0.79, 0.89)	< 0.001	40.1%	72.5%	87.3%

CEA  =  carcinoem-bryonic antigen; CYFRA21-1  =  cytokeratin 21–1; DLCO  =  diffusing capacity for carbon monoxide.

**Table 5:** Development of a clinical scoring system to predict survival of interstitial pneumonia characterized by UIP in HRCT.

Variables	Cut-off value	Score
**A**		
Age	≤ 66.5 years	0
	> 66.5 years	1
**C**		
CEA	≤ 2.3 ng/ml	0
	> 2.3 ng/ml	1
CYFRA21-1	≤ 4.3 ng/ml	0
	> 4.3 ng/ml	1
**D**		
DLCO% predicted	> 40.1%	0
	≤ 40.1%	1
**S**		
Smoking history	No	0
	Yes	1

CEA  =  carcinoem-bryonic antigen; CYFRA21-1  =  cytokeratin 21–1; DLCO  =  diffusing capacity for carbon monoxide.

### Association of clinical scoring system with survival of patients with fibrosing ILD in the validation cohort

**Figure 2:**
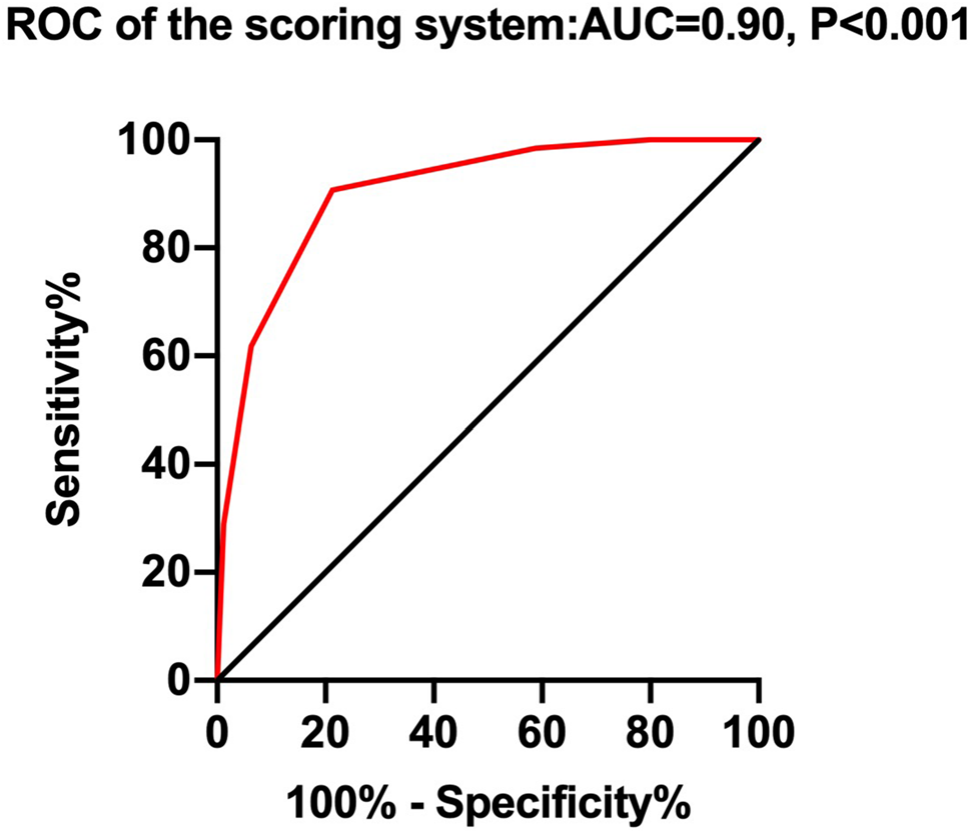
ROC curve of the scoring system for predicting survival of fibrosing ILD in the derivation cohort.

**Figure 3:**
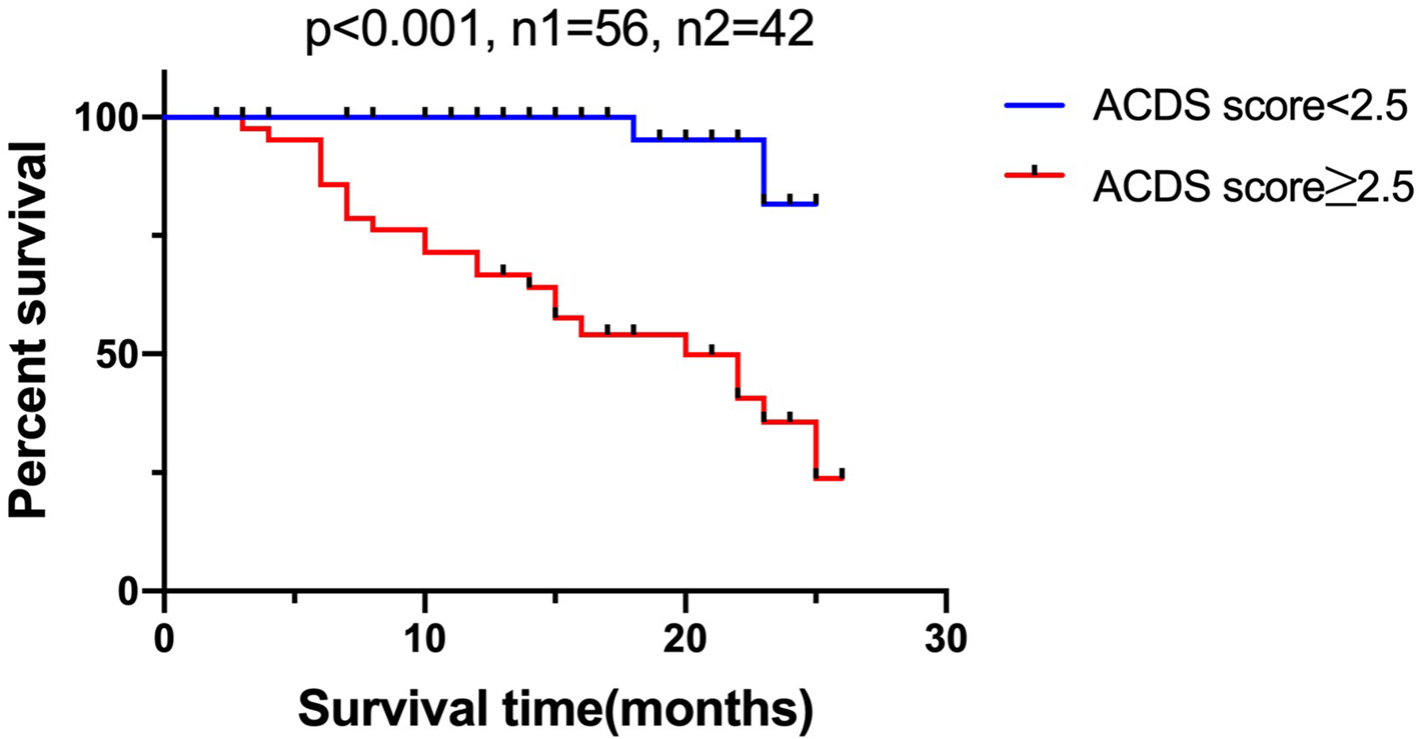
The respective Kaplan–Meier curve of fibrosing ILD patients with lower ACDS score group and higher ACDS score group in the validation cohort.

ROC curve was calculated to compare the predictive value of the scoring system in the derivation cohort. The ROC curve was shown in Fig. 2. The area under the curve of the scoring system for predicting survival of fibrosing ILD was 0.90 (95%CI: 0.87–0.94, *P* < 0.001). The cutoff value was 2.5 with their corresponding specificity (90.7%) and sensitivity (78.8%). In the validation cohort, the patients were divided into a higher ACDS score group (n = 42, ACDS score ≥ 2.5) and a lower ACDS score group (n = 56, ACDS score < 2.5) to analyze the survival using the Kaplan–Meier method (Fig. 3). The log-rank test showed a significant difference in survival between the two groups (*p* < 0.001).

## Discussion

The present study retrospectively compared the clinical and follow-up data between 259 survivors and 80 decedents with fibrosing ILD in the derivation cohort. In this study, we demonstrated that smoking history, age, CEA, CYFRA21-1 and DLCO% predicted could predict the survival of fibrosing ILD patients independently. A new predictive scoring system namely ACDS (age [A], CEA and CYFRA21-1 [C], DLCO% predicted [D], and smoking history [S]) was proposed. Importantly, we found that scoring system level was closely associated with the prognosis of fibrosing ILD patients. Furthermore, we demonstrated that patients with relatively low ACDS score had significantly longer overall survival than patients with relatively high ACDS score in the validation cohort.

Fibrosing ILD had similar biological and clinical behaviours which was characterised by progressive deterioration in lung function, progressive deterioration in lung function and high mortality rate^12,13^. Investigating the prognostic value of markers across fibrosing ILD was of great importance to clinical evaluation and make continues to elucidate the approach to fibrosing ILD management. In the past few years, several serum markers were identified as simple and readily accessible biomarkers to predict the survival and severity of fibrosing ILD. There were researches studying tumor markers such as CEA , carbohydrate antigen 19–9 (CA 19–9) and CYFRA21-1 that might reflect the severity and prognosis of fibrosing ILD^14–16^. One retrospective study by Fahim A et al., which included 41 non-smoking patients with idiopathic pulmonary fibrosis(IPF), reported that serum CEA concentration was elevated in approximately half of patients with IPF and was correlated with disease severity^17^. These results were consistent with the finding of our study of CEA as a biomarker in fibrosing ILD patients. In our study, CEA was identified as an independent prognostic factor for fibrosing ILD. CEA is a glycoprotein involved in cell adhesion and is produced by colonic epithelium. It has reported that CEA localizes in metaplastic epithelium lining honeycombed bronchioles by immunohistochemical staining. As cuboidal pneumocytes are the predominant source of epithelial renewal in severe lung damage and fibrosis, these cells are the most likely source of CEA release^18^.

In this study, elevated serum levels of CYFRA21-1 were observed in decedents group with fibrosing ILD. In a study by Vercauteren et al., higher level of CYFRA 21–1 in BAL of IPF patients resulted in worse survival in comparison with the CYFRA 21–1 low counterpart^19^. The expression of CYFRA21-1 in the lung has been identified in bronchiolar epithelial cells and pneumocytes. Elevation of serum CYFRA21-1 concentration might be associated with lysis or regeneration of these cells^15^. Furthermore, we demonstrated that serum CEA and CYFRA21-1 were significantly correlated with decreased DLCO%predicted in this study. The severity of ILD is usually based on pulmonary function test results such as DLCO%predicted^20^. Thus, serum CEA and CYFRA21-1 levels might be useful for reflecting the severity of fibrosing ILD.

A large amount of studies reported that smoking was closely associated with the onset and progress of pulmonary fibrosis^21,22^. A possible explanation may be that cigarettes contain the cytotoxic, mutagenic and proinflammatory substances. According to previous reports, these substances caused cellular oxidative stress, increased epithelial cell apoptosis, and dysregulation of immune responses, which was responsible for the progress of pulmonary fibrosis^23,24^. In addition, smoking affects the function of macrophages. It induced macrophage polarization to M2 phenotype that enhance the regression of inflammation and tissue remodeling^25^. Therefore, smoking cessation could be a good way to slow down the development of pulmonary fibrosis in the patients with ILD.

In the past years, few models has been proposed to predict the severity and prognosis of IPF. Glasgow prognostic score (GPS) has been reported to play an important role in predicting mortality in patients with acute exacerbation of IPF^26^. In our study, smoking history, age, CEA, CYFRA21-1 and DLCO% predicted were identified as independent factors for predicting the prognosis of fibrosing ILD. Moreover, based on these variables, a new predictive scoring system namely ACDS (age [A], CEA and CYFRA21-1 [C], DLCO% predicted [D], and smoking history [S]) was proposed. The scoring system was demonstrated to be as a predictive value for the survival of fibrosing ILD. However, it still needs further perspective study to verify the power of this scoring system based on multicenter and large population of fibrosing ILD patients.

Some limitations of this study should be noted. First, this was a retrospective and observational study of data obtained from a single center. In addition, the mechanism underlying the association of each biomarker with fibrosing ILD remains to be clarified in further in vivo and in vitro studies.

## Conclusions

In conclusion, smoking history, age, CEA, CYFRA21-1 and DLCO% predicted were independent predictors of the prognosis of fibrosing ILD patients that offers the advantages of convenience, ease of accessibility and low cost. A new predictive scoring system namely ACDS may help predict prognosis in patients with fibrosing ILD.

## Data Availability

The datasets used and/or analysed during the current study available from the corresponding author on reasonable request.
